# Modeling Public Opinion Reversal Process with the Considerations of External Intervention Information and Individual Internal Characteristics

**DOI:** 10.3390/healthcare8020160

**Published:** 2020-06-05

**Authors:** Tinggui Chen, Yulong Wang, Jianjun Yang, Guodong Cong

**Affiliations:** 1 School of Statistics and Mathematics, Zhejiang Gongshang University, Hangzhou 310018, China; 2 School of Management and E-Business, Zhejiang Gongshang University, Hangzhou 310018, China; yulong1105@yeah.net; 3 Department of Computer Science and Information Systems, University of North Georgia, Oakwood, GA 30566, USA; Jianjun.Yang@ung.edu; 4 School of Tourism and Urban-Rural Planning, Zhejiang Gongshang University, Hangzhou 310018, China; cgd@mail.zjgsu.edu.cn

**Keywords:** public opinion reversal, public opinion polarization, external intervention information, individual internal characteristics

## Abstract

With the rapid development of “we media” technology, external information about the same sudden hot social event is often involved repetitiously, leading to frequent public opinion reversal. However, the phenomenon of public opinion reversal process usually has a long-lasting duration and spreads wide, making the event itself attract the widespread attention of ordinary people. Focusing on the public opinion reversal process of sudden social hot topic (a popular and widely discussed issue), this paper firstly identifies the internal and external factors that affect the reversal, namely individual internal characteristics and external intervention information. Secondly, information intensity and the amount of information perceived by individuals are introduced to describe the impact of external intervention information on the public opinion reversal. Thirdly, the parameters of individual attention and conservation are used to describe the process of individual’s selection of external information, so as to reveal the influence of the internal characteristics on public opinion reversal, and then build a public opinion reversal model. Fourthly, the effects of information intensity and individual attention, as well as individual conservation on the process of public opinion reversal are analyzed by simulation experiment. Simulation results show that: (1) the intensity of external intervention information affects the direction and degree of public opinion reversal; (2) when individual conservation is strong or individual attention is weak, even if external intervention information is strong, there will still be no obvious reversal of public opinion. Subsequently, the rationality and effectiveness of the proposed model are verified by a real case. Finally, some recommendations and policy implications are also given.

## 1. Introduction

After a sudden social hot event, which refers to the events that cause concern among the masses at a certain period of time [1], the information will spread rapidly through the network platforms, and people will generate an initial viewpoint according to the information obtained, and communicate with others through network forums, discussion groups, etc. However, the dispute or affirmation of different views will lead to a climax. With the continuous intervention of external information, the attitude of netizens towards the event changes accordingly [2], which leads to public opinion reversal. Usually, the network public opinion reversal phenomenon has a long-lasting duration and spreads widely. For example: “Three Times the Support Rate Reversed During the 2016 US Election”, and “Kanye &Taylor Recording Event” attracted the continuous attention of netizens, and caused a spate of network violence, impacting social harmony and stability. Based on this, the study on the mechanism of the public opinion reversal has important theoretical and practical significance.

At present, studies are rare on public opinion reversal in academia. The main method is qualitative analysis of the phenomenon with the combination of psychology [3], communication science and other relevant disciplines. Some scholars also conduct quantitative research through modeling and simulation [4]. However, the qualitative method is mainly based on personal experience, and analyzes the causes, characteristics and coping strategies associated with public opinion reversal, which is affected by subjectivity. However, quantitative methods most likely use external factors, such as intervention mechanisms to analyze the causes of public opinion reversal, without considering the internal causes of public opinion reversal from the perspective of individual internal characteristics. In fact, only by fully studying the internal and external driving forces of public opinion reversal can we understand the internal mechanism at a deeper level. Therefore, this paper will reveal the evolutionary mechanism of public opinion reversal from internal and external affected factors, namely, external intervention information and individual internal characteristics.

Generally speaking, when the external intervention information is more positive, the individual attitude will become positive. However, when the external intervention information is relatively negative, the individual attitude will also become negative under the influence of the external intervention information [5]. Therefore, the influence of the external intervention information is an important factor for the formation of public opinion reversal. In addition, the key to whether an individual’s attitude will continue to change when influenced by external information lies in individual internal characteristics, which include individual attention to the event, the degree of individual psychological conservation, and the interaction pattern between individuals. Based on the fact, this paper introduces the parameters of information intensity, individual attention and conservation, considers the internal and external factors affecting public opinion reversal, and constructs a network public opinion reversal model. Finally, the influences of different information intensity, individual attention and individual conservation, as well as interaction characteristics on the phenomenon of public opinion reversal, are explored by experimental simulation. The rationality and effectiveness of the model proposed in this paper are verified by combining with a real case as well.

## 2. Literature Review

The frequent occurrence of the public opinion reversal has aroused the wide attention of many scholars. Research on this phenomenon mainly focuses on the following two aspects: one is public opinion polarization; the second is the public opinion reversal.

Public opinion polarization is usually considered an important stage of public opinion reversal, which is formed on the basis of the influence of external intervention information and the internal characteristics of individuals. At present, many scholars have conducted in-depth research on public opinion polarization. For example, Chen et al. [6] studied the impact of individual heterogeneity and introduced dynamic conformity. Gabbay et al. [7] presented a novel explanation for the group polarization effect, whereby discussion among like-minded individuals induces shifts toward the extreme. Matakos et al. [8] considered the problem of measuring and reducing polarization of opinions in a social network, using a standard opinion formation model to define the polarization index, and captured the tendency of opinions to concentrate in network communities. Kleiner et al. [9] introduced a new index to measure public opinion polarization, and examined whether ideological extremism makes individuals more susceptible to environmental opinion polarization. Li and Xiao [10] proposed a multidimensional opinion evolution model for studying the dynamics of opinion polarization, which demonstrated that the evolution processes in different dimensions of opinion showed correlation under certain specific conditions. Del et al. [11] explored the structure of this evolution by taking users’ emotion and comments into account. Experimental results showed that users on Facebook tended to select information that adhered to their system of beliefs, and to form polarized groups. In addition, by introducing the social preference theory, Chen et al. [12] revealed the micro-interaction mechanism of public opinion polarization, and showed that different social preferences held by individuals had different influences on the public opinion polarization effects. Research on public opinion polarization in the above-mentioned literature mainly focuses on the analysis of general law, but rarely discusses the subsequent reversal process. In fact, after public opinion polarization, public opinion reversal may take place. Due to its complexity and various affecting factors, there are relatively few studies on public opinion reversal. However, public opinion polarization is the basis for the formation of public opinion reversal, which is usually the result of further evolution. Based on this, in-depth research on public opinion polarization is required to reveal the mechanisms of public opinion reversal.

Some scholars have conducted the research regarding public opinion reversal. For instance, Huang et al. [13] introduced a new model, which took the relationship between individual cognitive bias and their corresponding choice behavior into account, and pointed out that cognitive bias and the change ratio of choice behavior dominated the probability of public opinion reversal. Zhu and Hu [14] analyzed information strength, time of releasing information and different types of counter information through a large number of simulation experiments, to find out the principles of public opinion reversal. Schmidt et al. [15] Evaluated Facebook users’ opinion polarization on the topic of vaccination, and made the conclusion that the introduction of dissenting information into a sub-group was disregarded, and could produce a backfire effect, thus reinforcing the pre-existing opinions within the sub-group. Yan and Jiang [16] investigated the competitive spreading phenomenon by comparing positive and negative information, and simulation results of the model showed that a group in a competitive spreading system could not absolutely win, no matter whether this group spread positive or negative information. Flache et al. [17] found that a renegade minority (‘out group-lovers’) could have a key role in avoiding mutually negative inter-group relations and even elicited ‘attitude reversal’. Deng et al. [18] found that a positive messages could influence the opinion dynamics in the early stages of opinion formation, and then decided the opinion formation like a butterfly effect. Lewandowsky et al. [19] explored when a minority of agents that was resistant to the evidence was introduced for scientific discussion, the simulated scientific community still acquired firm knowledge, but consensus formation was delayed. Zhao et al. [20] analyzed the impacts of the opinion leaders and the environmental noises on the final opinions of the opinion followers. The simulation results showed that, as the variances of the environmental noises increased to a certain threshold, the followers were influenced strongly by the opinion leaders. Zan et al. [21] considered the counterattack mechanism of rumors spreading, added counterattack individuals to the classical Susceptible Infected Recovered (SIR) model, and introduced the Susceptible Infective Counterattack Refractory (SICR) model and the adjusted-SICR model. The refuting–persuading mechanism in the SICR model reflected the self-resistance characteristic of networks to a rumor. Gargiulo and Gandica [22] introduced a tunable external media pressure, finding that the combination of homophily and media made the media effect less effective, and led to strongly polarized opinion clusters. Research on public opinion reversal in the above literature mainly focuses on the simulation of external factors, or the simulation of the evolution process of the phenomenon of public opinion reversal by building a model. However, there are relatively few literatures that comprehensively consider the internal and external factors affecting public opinion reversal. Due to its complexity and many influencing factors, it is difficult to make a quantitative analysis on reversal process. However, public opinion reversal is usually affected by external intervention information and individual internal characteristics. Therefore, this paper discusses the process of public opinion reversal from two aspects: external information intervention and the influence of individual internal characteristics.

To sum up, most studies on public opinion reversal analyze its causes from the perspective of external influencing factors or intervention mechanism, but few studies analyze the evolutionary mechanism of public opinion reversal from the perspective of internal and external aspects. In fact, the external intervention information and individual internal characteristics are the dual driving forces to promote the public opinion reversal, and the dual influences are the key to the continuous deflection or reversal of public opinion, and the current research on the public opinion reversal combining two factors is still rare in the literature. Generally, in the evolution process of public opinions, affected by the constant release of external information and individual internal characteristics, individual attitudes will change under the dual actions of internal and external factors, and thus promote the continuous deflection or even reversal of public opinions. In general, when the external information is released, individuals will first acquire and perceive external information with a combination of attention degree, then select the relevant external information by comparing their various psychological expectations (individual conservative degree), and then realize the external information transmission through interaction with close friend’s viewpoint. Therefore, this paper firstly introduces the amount of information perceived that prompts individuals to reflect on the influence of external intervention information on themselves, and then introduces individual attention and individual conservation to reflect the influence of individual internal characteristics on attitudes, so as to build a public opinion reversal model and study the internal mechanism of its evolution. Finally, the influences of different factors, such as information intensity, individual attention and conservation on the process of public opinion reversal are analyzed, in combination with the simulation experiment, and the rationality and effectiveness of the model are verified by a real case.

## 3. Model Construction

**Figure 1:**
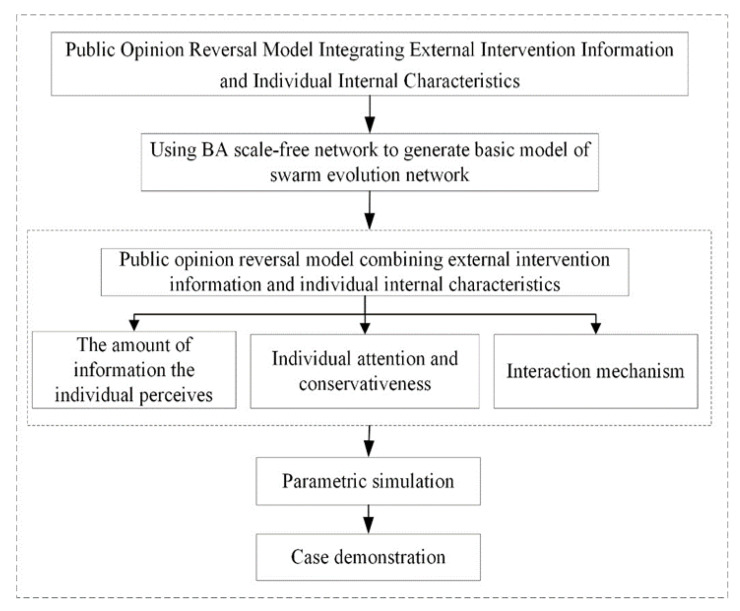
Study framework.

Based on the multi-agent method proposed by Monte Carlo, this paper uses agent to represent the individual in the network, and the network size is set as *N*, that is, there are *N* netizen nodes in the network. The individual attitude value is represented by any number in the interval of [−1,1], that is, individuals in the interval of [−1,0] have a negative attitude, while individuals in the interval of [0,1] have a positive attitude. The study framework of the paper is shown in Figure 1.

Agent *i*, after releasing the external intervention information, will acquire and perceive related information, so as to form the corresponding amount of information the individual perceives. Subsequently, agent *i* chooses external information by comparing individual conservation, and spreads information during interactions with close friends. Therefore, affected by the constant change of external information and individual internal characteristics, the attitude of agent *i* towards events keeps changing, and then public opinion reverses. Based on this, this paper builds a model of public opinion reversal based on the internal and external factors. The parameters and variables involved in the model are shown in Table 1 and Table 2.

### 3.1. Individual Perception $O_{i}^{n}$

The influence of external intervention information contributes to public opinion reversal. Generally, with the release of different external information, individuals will perceive the viewpoint tendency conveyed by this external information, which is defined as the amount of information the individual perceives, $O_{i}^{n}$. On the one hand, the amount of information the individual perceived is affected by external information, on the other hand, it is affected by individual attention to the event itself. Based on this, its specific expression is as follows.

(1) $$O_{i}^{n}=\alpha_{i}*\left(\mathrm{sgn}\left(I^{n}\right)*I^{n}*\left(\mathrm{sgn}\left(I^{n}\right)-{\dot{x}}_{i}^{n-1}\right)\right)\cdot (n\;=\;1,2,3\ldots )$$

where $I^{n}*\left(\mathrm{sgn}\left(I^{n}\right)-{\dot{x}}_{i}^{n-1}\right)$ reflects the influence of external information on agent *i*, and ${\mathrm{sgn}(I}^{n})$ is used to control the polarity of external information. The influence degree of external information will be constrained by *α_i_*(*α_i_* ∈ [0,1]). When *α_i_* = 0, it means that individual attention is 0, and $O_{i}^{n}$ is 0. When 0 < *α_i_* < 1, $O_{i}^{n}$ increases with the increase of *α_i_.*$O_{i}^{n}\in \left[0,2\right]$, which is used to ensure the attitude reversal of some extreme individuals. For example, the initial attitude value of an extreme individual is −1. When the perceived information amount $O_{i}^{n}$ is 2, the attitude value will become 1. We call this individual an extremely swinging individual, and this phenomenon occurs from time to time in the real world.

#### 3.1.1. Information Intensity *I^n^*

**Figure 2:**
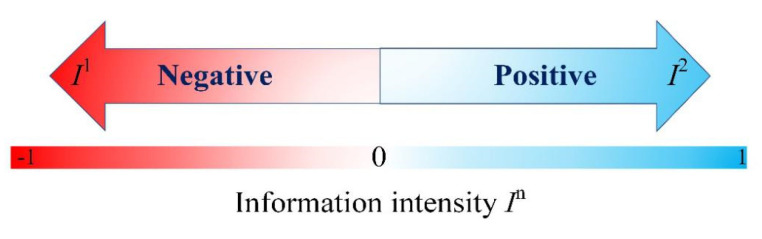
*I^n^* diagram.

In the process of spreading public opinion, the influence of external intervention information includes two aspects: the strength of its influence and the tendency of the viewpoint. In order to better describe it, the vector value *I^n^* is introduced to define the information intensity (*I^n^* ∈ [−1,1]), which represents the influence of *n* kinds of external intervention information. As shown in Figure 2, *I*^1^ and *I*^2^ represent two kinds of external information with different directions and strengths, respectively.

In real life, the greater the influence of media is, the greater the probability of individuals paying attention to the media information will be [23]. Therefore, the probabilistic influence of media influence on individual attention to their information is described in a probabilistic way. Assuming that the probability of agent *i*’s perception of external information is *p_i_*, which changes with external information intensity *I^n^*. The greater the value of information intensity *I^n^* is, the greater the probability of individuals’ perception is. Therefore, *p_i_* can be defined as follows:

(2) $$p_{i}=1-\frac{{1\;-\;|I}^{n}|}{{1+|I}^{n}|}$$

where *p_i_* increases with the increase of |*I^n^*|. When |*I^n^*| = 0, the influence of external information is 0, and *p_i_* = 0. When |*I^n^*| = 1, the influence of external information is 1, and *p_i_* = 1.

#### 3.1.2. Individual Attention *α*

Due to the differences in people’s social background, living environment, learning styles, various experiences and understanding, people will pay different attention to the same hot topics and social events, which reflects individuals’ heterogeneous characteristics. In the process of the public opinion evolution, this mainly plays two roles: firstly, when an individual pays more attention to a certain type of event, the individual will give stronger feedback to the external information of the relevant event; Secondly, when an individual pays less attention to a certain kind of event, they will not give feedback on such information, even if the external information of related events is strong. This individual heterogeneous feature is individual attention *α*(*α* ∈ [0,1]), which represents the degree of attention of an individual to a public opinion event. *α* = 0 indicates that the individual is not concerned about an event, while *α =* 1 indicates that the individual is extremely concerned about the event.

### 3.2. Individual Conservation k and Hesitation h

**Figure 3:**
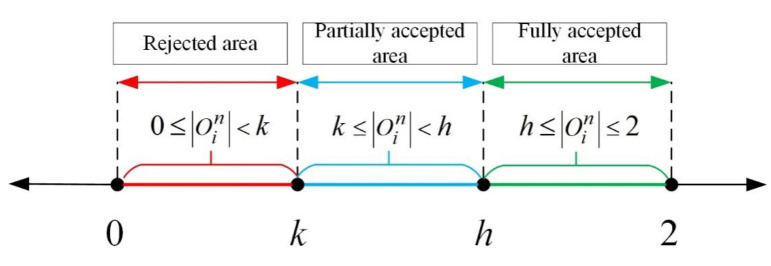
Individuals’ acceptance of external information.

Generally, people will constantly update their attitude value under the influence of new information and ideas [24]. However, differences between people determine that the acceptance of new information and viewpoints vary with individuals. After perceiving external information, people update and spread their own attitudes by comparing various psychological expectations. Social judgment theory [25] holds that people’s acceptance of new information and ideas can usually be divided into three parts: the reject area, the partially accepted area and the fully accepted area. In order to describe these three situations more reasonably, this paper introduces the concepts of degree for individuals’ conservation and hesitation to describe the process of individuals’ acceptance of external information, as shown in Figure 3.

From Figure 3, according to the threshold of an individual’s tendency to accept external information, the value can be divided into the aforementioned three areas. After that, through comparing the amount of information $O_{i}^{n}$ perceived by the individual with his conservation and hesitation, the individual will select whether to accept the viewpoint transmitted by the *n*th external information. Here, *k* is the individual’s conservation. Since $O_{i}^{n}$ ∈ [0,2], in order to make the individual’s conservation more in line with practical requirements. We suppose *k* ∈ [0,2]. *h* represents the individual’s hesitation (*h* ∈ [0,2]). The smaller its value is, the higher the individual’s enthusiasm to accept and spread information [26] will be. The specific calculation process is shown as follows:

(3) $$x_{i}^{n}=\{\begin{array}{c} {\dot{x}}_{i}^{n-1},if\;0\;⩽\;\left|O_{i}^{n}\right|\;<\;k\\ {\dot{x}}_{i}^{n-1}{+O}_{i}^{n}\;*\;\frac{{h-O}_{i}^{n}}{h-k},\;if\;k\;⩽\;\left|O_{i}^{n}\right|\;<\;h\\ {\dot{x}}_{i}^{n-1}{+O}_{i}^{n},\;other \end{array}$$

When $0\;⩽\;\left|O_{i}^{n}\right|\;<\;k$,${\;O}_{i}^{n}$ of agent *i* falls into reject area, agent *i* does not update attitude value. When $k\;⩽\;|O_{i}^{n}|\;<\;h$,${\;O}_{i}^{n}$ of agent *i* falls into the partially accepted area, agent *i* updates their attitude value according to Formula (3). In other cases, $O_{i}^{n}$ of agent *i* falls into the fully accepted area, so agent *i* updates their attitude value according to Formula (3).

### 3.3. Interaction Rule

Due to the social characteristics, netizens often hope to be recognized by other individuals through interactive behaviors after the formation or transformation of their views. In this process, while influencing others’ views, their own views will also be changed to a certain extent. In order to describe this process, the interaction model of individual views is constructed based on the Jager–Amblard (J–A) model [27].

#### 3.3.1. Intimacy, *q*, and the Average Attitude Value of Node *j′*,${\;X}_{j^{\prime }}^{n}$

Individual interactions tend to occur between close friends, and the probability of communication between estranged people is relatively small. Based on this, this section defines the relationship degree between nodes by the intimacy index. The shortest distance connecting nodes *i* and *j* in the network is *d_ij_*, and the exponential form of *d_ij_* is the intimacy between nodes, which is expressed by Formula (4):

(4) $$q_{ij}{=e}^{{1-d}_{ij}}$$

According to Formula (4), if the distance between two nodes in the network is longer, the intimacy degree is lower, and the greater the distance between them, the faster the attenuation of the intimacy degree is. When *d_ij_* = 1, then the $q_{ij}\;$= 1. Generally speaking, individuals usually choose close friends to interact with their views. Therefore, in the interaction model, agent *i* only chooses those *j′* as its interaction nodes whose intimacy is 1. The average attitude value $X_{j^{\prime }}^{n}\;$of these interaction nodes is calculated, as shown in Formula (5).

(5) $$X_{j^{\prime }}^{n}\left(t\right)=\frac{\sum^{\;}x_{j^{\prime }}^{n}\left(t\right)}{\sum^{\;}j^{\prime }}$$

#### 3.3.2. The Setting of Interaction Rules

During the interaction of individual views, individual *i* will update their own attitude value through comparing it with the average attitude value $X_{j^{\prime }}^{n}$ of the selected interaction node. When $x_{i}^{n}$ is close to $X_{j^{\prime }}^{n}$ (in the assimilation effect zone), agent *i* will be encouraged to enhance their own attitude. When their attitude values are quite different (in the repulsion effect zone), agent *i* will weaken their attitude, due to the surrounding pressure. At moment *t*, agent *i* adjusts the attitude of the next moment according to the average attitude value of the interaction node, and updates rules according to different value as follows:

(1) When $\left|x_{i}^{n}\;{-\;X}_{j^{\prime }}^{n}\right|{<\;d}_{1}$,

(6) $${\dot{x}}_{i}^{n}{(t+1)=x}_{i}^{n}{(t)+\mu (x}_{i}^{n}{(t)\;-\;X}_{j^{\prime }}^{n}\left(t)\right)$$

where *µ* is assimilation parameter, *µ* ∈ (0,0.5).

(2) When $\left|x_{i}^{n}{-X}_{j^{\prime }}^{n}\right|{>\;d}_{2}$,

(7) $${\dot{x}}_{i}^{n}\left(t+1\right){=x}_{i}^{n}\left(t\right){-\;\beta (x}_{i}^{n}{(t)\;-\;X}_{j^{\prime }}^{n}\left(t)\right)$$

where β is divergence parameter

(3) In other cases, the attitude value of individual *i* remains unchanged, as follows:

(8) $${\dot{x}}_{i}^{n}\left(t+1\right){=x}_{i}^{n}\left(t\right)$$

**Figure 4:**
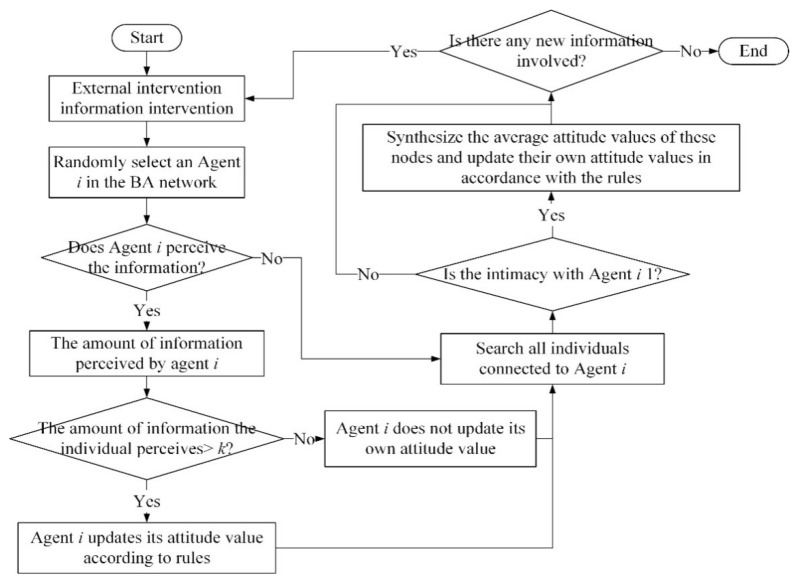
Simulation diagram based on the multi-agent method proposed by Monte Carlo.

According to the above analysis, the specific simulation process of this paper is shown in Figure 4.

## 4. Simulation Experiment

In this section, we use the commercial mathematics software MATLAB, produced by the American Math Works company for simulation experiments (Natick, MA, USA). Combined with the public opinion reversal model, this section discusses the impacts of information intensity, individual attention, conservation and hesitation, interaction mode and network topology structure on the public opinion reversal process, so as to reveal its internal evolutionary mechanism.

### 4.1. The Impact of Information Intensity on Opinion Reversal

Combined with the public opinion reversal model, the initial parameters of the model evolution are set. Assuming that the information intensity under the initial state is 0, indicating that the hot social events have not occurred yet. In addition, the initial attitude value $x_{i}^{0}$ of agent *I* represents its empirical attitude value for previous similar events, which follows a normal distribution of *N*~(0,0.4) and maps to the interval [−1,1]. The attitude value less than −1 is set to −1, while the attitude value greater than 1 is set to 1. In this way, the initial attitudes of most individuals are relatively neutral, with a few individuals holding extreme attitudes. The hypothesis is consistent with the distribution of the group’s attitude towards certain events in the real world.

In order to better describe the distribution of extreme attitude in groups, the individual attitude greater than 0.9 or less than −0.9 is defined as individual attitude polarization, namely negative attitude polarization and positive attitude polarization, respectively. The proportion of polarized individuals in the group is polarizability, which can be further subdivided into positive polarizability and negative polarizability. The positive polarizability represents the proportion of positive polarized individual in the group, and negative polarizability represents the proportion of negative polarized individual in the group. The simulation network is constructed based on the Barabasi–Albert (BA) scale-free network [28], and the node size is set to 500. Considering the comprehensive visualization, other parameters are set as: *d*_1_ = 0.3, *d*_2_ = 0.3, *u* = 0.3, *β* = 0.2, *α* = 1, *k* = 0.1, *h* = 0.2, evolution time *T* = 50.

**Figure 5:**
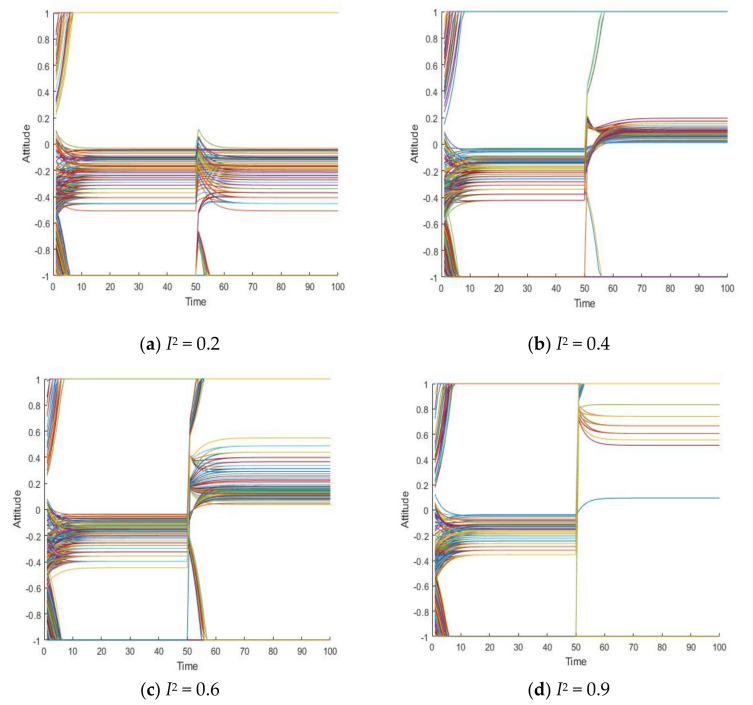
Group attitude evolution graph.

**Figure 6:**
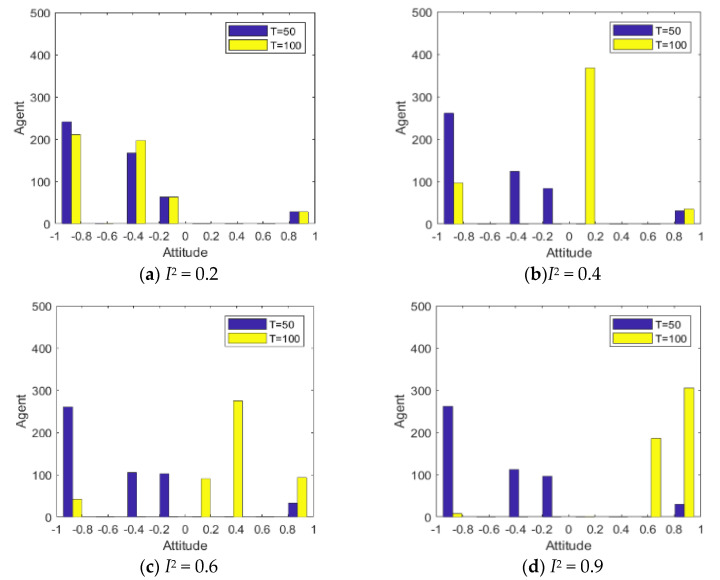
Group attitude distribution histogram.

**Figure 7:**
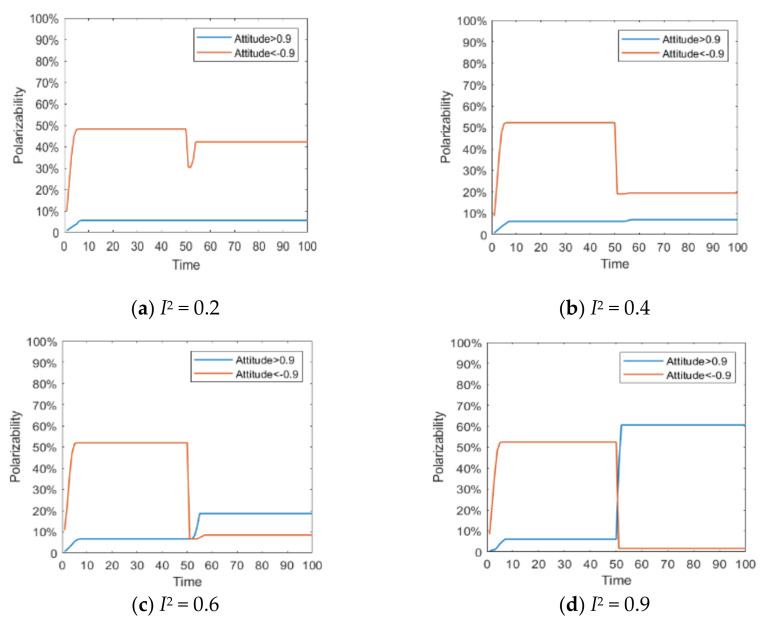
Polarization curve.

Since the literature [14] has conducted in-depth discussions on the influence of external information on public opinion reversal, this paper only briefly expounds the influence of external intervention information. The information in the public opinion field usually has a certain extent of “untruth”, so the truth of the event often needs various evidences. When the external intervention information is released, individuals will discuss the relevant information and form a consensus, that is, the public opinion polarization. With the release of a new round of messages and the influence of individual’s internal characteristics, individuals will launch a new round of discussion, affecting or reversing the original consensus of views, that is, the public opinion reversal. Therefore, it is necessary to study the reversal process after the public opinion polarization. Here, the information intensity of the initial information is set *I*^1^ = −0.6, and the information intensity *I*^2^ of the secondary information is 0.2, 0.4, 0.6 and 0.9, respectively. The specific simulation results are shown in Figure 5, Figure 6 and Figure 7.

Figure 5, Figure 6 and Figure 7 represent the group attitude evolution graph, group attitude distribution histogram and polarization curve when *I*^1^ = −0.6, and *I*^2^ = 0.2, 0.4, 0.6 and 0.9, respectively. From above figures, when *I*^1^ = −0.6, group attitude polarization is formed, with the involvement of the secondary information, the attitude is changed to different extent. When *I*^2^ is small, group attitude is changed slightly, the overall attitude tendency has not changed significantly, but a few individuals have strengthened their own original attitude tendency. However, with the gradual enhancement of *I*^2^, the attitude of the vast majority of individuals tends to change, along with the direction of secondary information, and the attitude of some neutral individuals are first affected by it, indicating that the influence process of secondary information has a certain asymptotic property. In addition, with the gradual enhancement of the intensity of secondary information, the negative polarization decreases continuously, and the positive polarization increases gradually, and the vast majority of individuals in the group support the tendency viewpoint conveyed by the secondary information. At this time, the completely opposite polarization effect “one positive and one negative” is formed in the two periods of the evolution of public opinion (about *T* = 50) and the network public opinion is obviously reversed.

**Figure 8:**
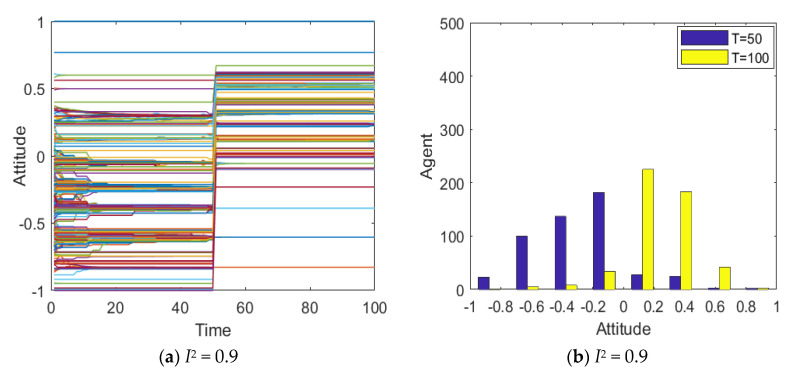
Simulation group opinion evolution chart and opinion distribution histogram based on the literature [14].

Figure 8 represents the simulated group opinion evolution diagram and group attitude distribution histogram based on the literature [14]. As can be seen from Figure 8, under the condition that other simulation conditions are consistent, when *I*^1^ = −0.6, the group attitude is under the influence of negative information, the attitudes of most individuals are between −0.6~0, the group attitude is relatively negative, but it has not formed an obvious negative polarization phenomenon. With the intervention of secondary information, under the influence of strong positive information, most individuals start to develop positive attitudes, and only a few individuals maintain negative attitudes, but individual attitudes mostly range from 0 to 0.6, and no positive polarization phenomenon is formed. To sum up, it can be found that, although the model proposed in the literature [14] simulates the phenomenon of public opinion reversal, it fails to truly reflect the evolution of attitudes of extreme individuals, so it is impossible to describe the phenomenon of reversed public opinion in reality. Contrarily, the model mentioned in this paper not only simulates the phenomenon of public opinion reversal, but also reasonably reflects the distribution and changes of extreme individuals’ attitudes, which is more in line with reality.

### 4.2. The Influence of Individual Attention on the Process of Public Opinion Reversal

The degree of individual’s attention to social hot events is often affected by two aspects: the first is the complexity of the event itself affecting the potential development of the event while the second is the degree of relevance to people’s lives, that is, whether the event itself is closely related to the lives of netizens. Generally speaking, the more complex the incident is and the more relevant it is to the public’s life, the more attention will be paid by netizens. For example, the support rate reversed during the 2016 US election aroused the public’s concern for a long time, due to its relevance to civil rights and complexity.

#### 4.2.1. The Influence of Different Individual Attention on the Process of Public Opinion Reversal

In general, the more an individual pays attention to an event, the more sensitive the individual is to relevant external information, thus giving stronger information feedback and promoting the changes in the evolution process of public opinion. In order to describe the difference in individual attention to hot events, if *α* belongs to *N*~(0.1,0.4), and maps to [0,1], it indicates low attention; if *α* belongs to *N*~(0.9,0.4), and maps to [0,1], it indicates high attention; if *α* belongs to *N*~(0.5,0.4), and maps to [0,1], it indicates medium attention; if *α* belongs to *U*~[0,1], the opinion evolution status will be discussed with the same quantity of individuals with different attention degrees. Based on this, this section will discuss the influence of individual attention on the process of public opinion reversal. Other parameters are set as: *I*^1^ = −0.6, *I*^2^ = 0.8, *k* = 0.1, *h* = 0.2, *T* = 50.

**Figure 9:**
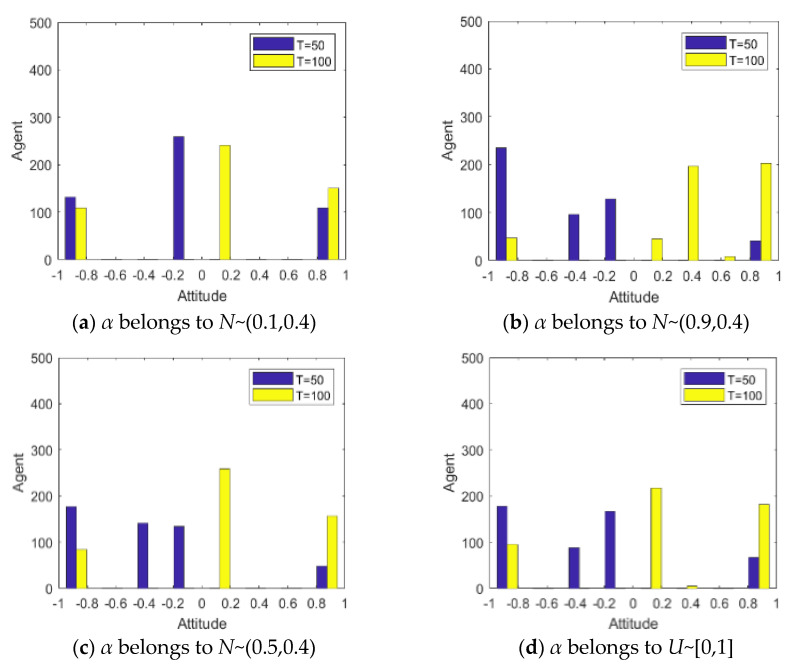
Histogram of group attitude polarization under different distribution of attention.

**Figure 10:**
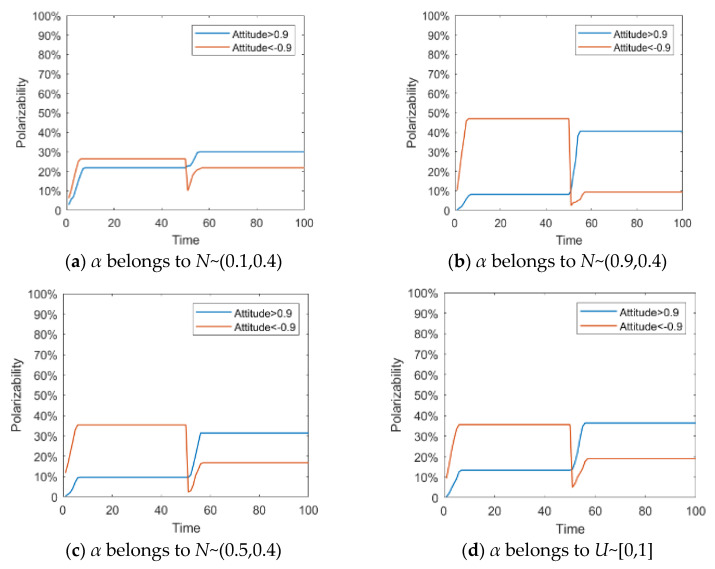
Curve of group attitude polarization under different distribution of attention.

Figure 9 and Figure 10 represent the histogram and curve of group attitude polarization under different distributions of attention, respectively. We can see from these two figures that the different distribution of individual attention can have an important impact on the public opinion reversal process. When *α* belongs to *N*~(0.1,0.4), the polarization of a group attitude does not change significantly, indicating that low individual attention gives less feedback. However, when *α* belongs to *N*~(0.9,0.4), the distribution and the polarization change significantly, leading to an obvious public opinion reversal. Thus, when individuals pay more attention to hot events, their feedback to external information will be more intense, and the group attitude will form a strong polarization and reversal phenomenon. However, when the attention degree of individuals in the group is generally or evenly distributed (*α* belongs to *N*~(0.5,0.4) and *U*~[0,1]), the group attitude will only slightly fluctuate with the guidance of external information, and then the public opinion will slightly reverse.

#### 4.2.2. The Influence of the Dynamic Change of Individual Attention on the Process of Public Opinion Reversal

The individual attention to an event is not fixed. Generally, with the development of the event and the gradual disclosure of the truth, the individual attention to public opinion events will change accordingly. In order to describe this process, it is assumed that individual attention follows different distribution at different times, and then the influence of its dynamic change on the process of public opinion reversal is analyzed. In this paper, the changes of individual attention to events are divided into four categories: (1) The degree of attention in the early stage is low, and gradually increases in the later stage (*T* < 50: *α* belongs to *N*~(0.1,0.4); *T* ≥ 50: *α* belongs to *N*~(0.9,0.4)); (2) The degree of attention in the early stage is general, and gradually enhances in the later stage (*T* < 50: *α* belongs to *N*~(0.5,0.4); *T* ≥ 50: *α* belongs to *N*~(0.9,0.4)); (3) The degree of attention in the early stage is high, and gradually decreases with the government intervention or other information interruption (*T* < 50: *α* belongs to *N*~(0.9,0.4); *T* ≥ 50: *α* belongs to *N*~(0.1,0.4)); (4) The degree of attention in the early stage is high, and continuously enhances in the later stage (*T* < 50: *α* belongs to *N*~(0.9,0.4); *T* ≥ 50: *α* belongs to *N*~(0.9,0.4)).

**Figure 11:**
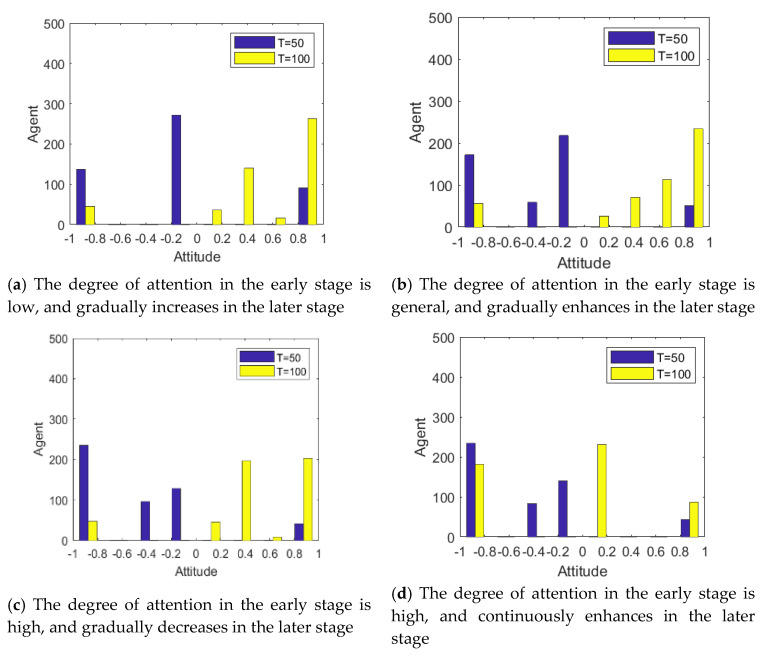
Histogram of group attitude with changes of individual attention.

**Figure 12:**
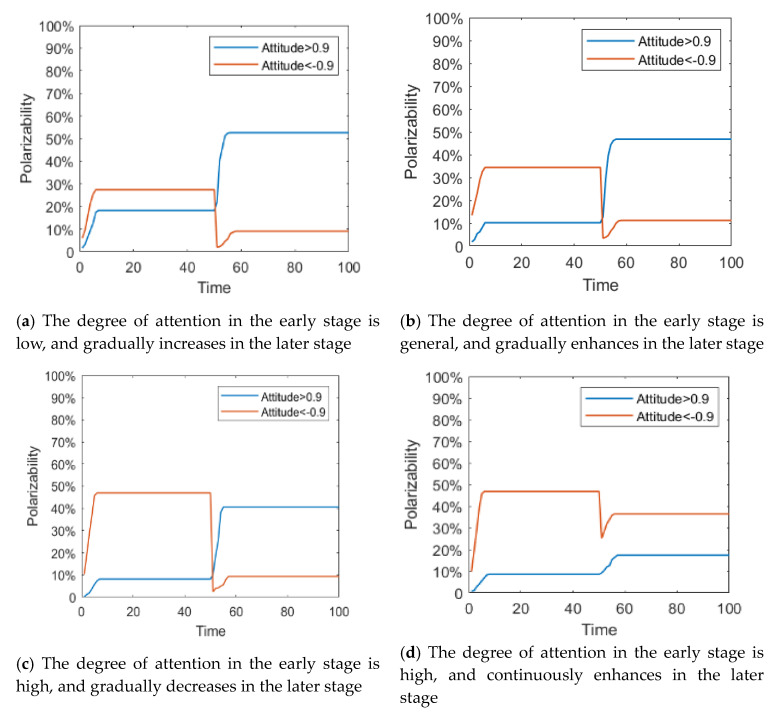
Curve of group attitude polarization with changes of individual attention.

Figure 11 and Figure 12 represent the histogram of group attitude and curve of group attitude polarization with changes of individual attention, respectively. These two figures show that when *α* remains relatively low at time = 0~50, the negative polarization rate is low. When *α* enhances at time = 50~100, positive polarization rates increased significantly, forming obvious public opinion reversal. When *α* is strong in the two time periods of evolution (About *T* = 50), the negative and positive polarization rates are both high, indicating that the group has a high degree of attitude polarization, and the two time periods form an obvious public opinion reversal. When *α* is strong first and then becomes weak, that is, *α* is strong at *T* = 0~50, and weak at *T* = 50~100, the negative polarization is higher. However, with declining individual attention, the positive polarization rate does not obviously change, which suggests that the volatility and reversal of public opinion can be avoided by distracting or reducing attention after spreading the hot issues.

### 4.3. The Influence of Individual Conservation and Hesitation on the Process of Public Opinion Reversal

Due to great differences in personality, behavioral willingness and other aspects, individuals will have different acceptance of new matters, information and ideas. Therefore, the reasons for individual attitude changes can be further explained through individual psychological conservation and hesitation, and then the internal motivation influencing public opinion reversal can be analyzed.

#### 4.3.1. The Influence of Static Distribution of Individual Conservation on the Process of Public Opinion Reversal

According to the conservation degree, individuals can be divided into three categories: individuals with weak degree of conservation (*k* ∈ [0,2/3]), individuals with general degree of conservation (*k* ∈ [2/3,4/3]), and individuals with strong degree of conservation (*k* ∈ [4/3,2]). The impact on public opinion reversal process is analyzed through the proportion of each kind in groups. Here, setting *I*^1^ = −0.6, *I*^2^ = 0.8, *T* = 50, *α* belongs to *N*~(0.9,0.4), and *h* belongs to *U*~[0,2].

**Figure 13:**
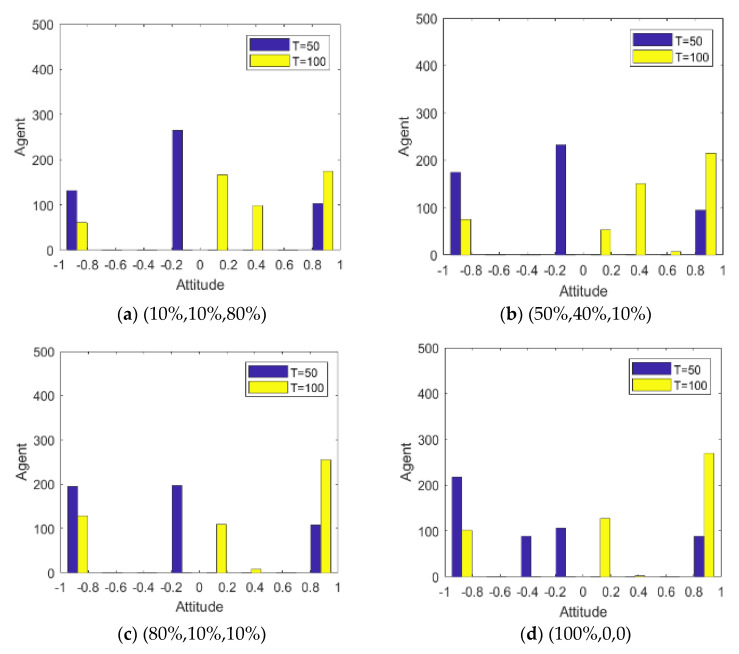
Histogram of the group attitude distribution under different proportion.

**Figure 14:**
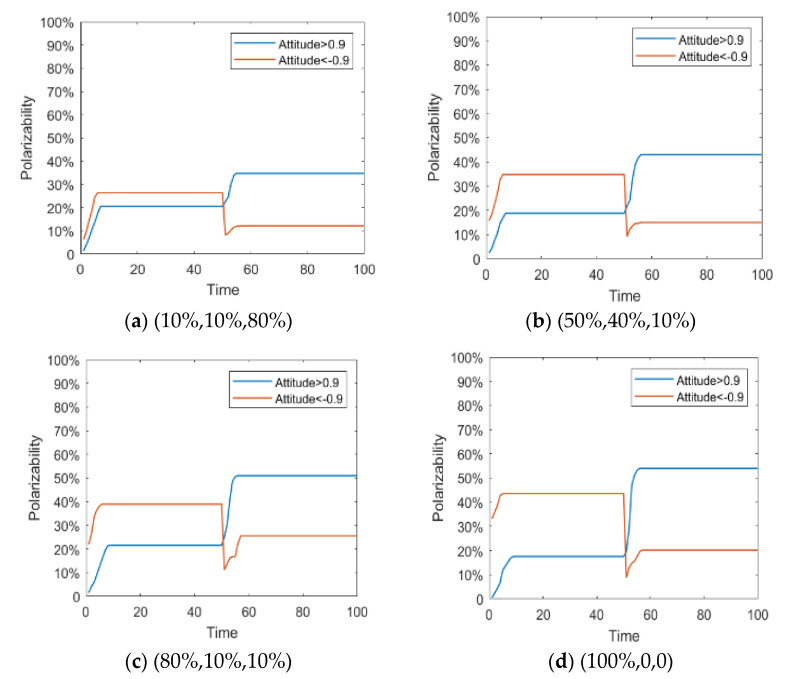
Curve of the group attitude polarization under different proportion.

Figure 13 and Figure 14 represent the histogram of the group attitude distribution and curve of the group attitude polarization under different proportion, respectively. From Figure 13 and Figure 14, when individuals with a strong conservative degree account for a large proportion of the group, the group attitude does not polarize or reverse. It means that a hot issue has failed to break through the individual psychological bottom line, and individuals are not interested to comment, so large-scale mass incidents will not occur. With the increase of the proportion of individuals with weak conservation in the group, the positive and negative polarization of the group attitude both increased, and an obvious public opinion reversal is formed around *T* = 50. This shows that, when some public opinion topics break through the psychological conservative bottom line of the public, the public’s enthusiasm for participation will be significantly enhanced, and then a strong polarization and reversal of public opinion will be formed.

#### 4.3.2. The Influence of the Dynamic Change of Individual Conservation on the Process of Public Opinion Reversal

With the gradual release of external information, individuals will gradually pay more attention to hot social events, and they will be more open to relevant external information. When information is released, individuals will be more willing to comment on the event itself, and this dynamic change in individual psychology will often play an important role in promoting the evolution of public opinion. Therefore, in order to describe the dynamic change of individual conservation, the following section studies the impact of dynamic change of individual conservation on the evolution of public opinion, by setting different proportions of three kinds of conservative individuals at different times.

**Figure 15:**
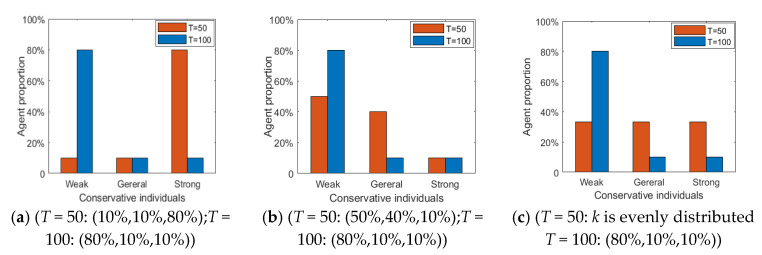
Distribution histogram of three kinds of individuals with different conservation at different times.

**Figure 16:**
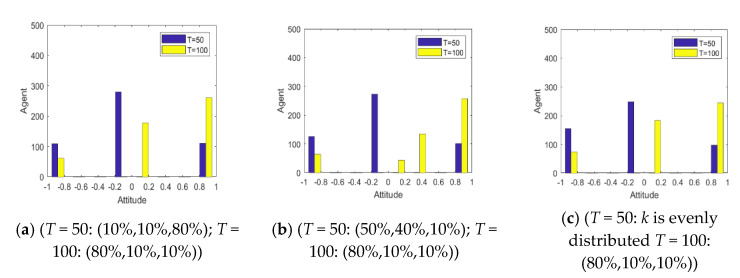
Distribution histogram of group attitude with different conservation proportion at different times.

**Figure 17:**
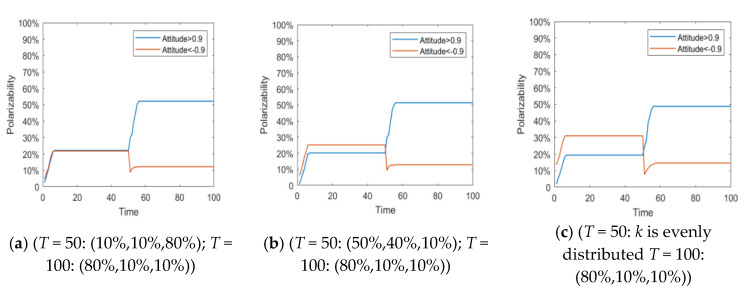
Curve of group attitude polarization with different conservation proportion at different times.

Figure 15 shows the distribution histogram of three kinds of individuals with different conservation at different time points. Figure 16 and Figure 17 represent the distribution histogram of group attitude and the curve of group attitude polarization with different conservation proportion at different times, respectively. According to Figure 16 and Figure 17, at *T* = 0~50, due to large proportion of weakly conservative individuals, the attitude distribution and polarization of the group do not change significantly from the initial state. However, with the increase of the proportion of weakly conservative individuals, the negative polarizability increases. At *T* = 50~100, due to large proportion of weakly conservative individuals, the positive polarization remains at a high level, and is not affected by extreme viewpoints. It indicates that, with the decreasing individual psychological conservatism degree, individuals are more willing to participate in the discussing relevant events, thus promoting the public opinion reversal. Therefore, while intervening in public opinion, the low degree of public participation in the early stage should not be ignored, which may arise at a larger scale of online public opinion at later stage.

#### 4.3.3. The Relation among Three Conservative Individuals, Hesitation Individuals and Attitude Polarization

**Figure 18:**
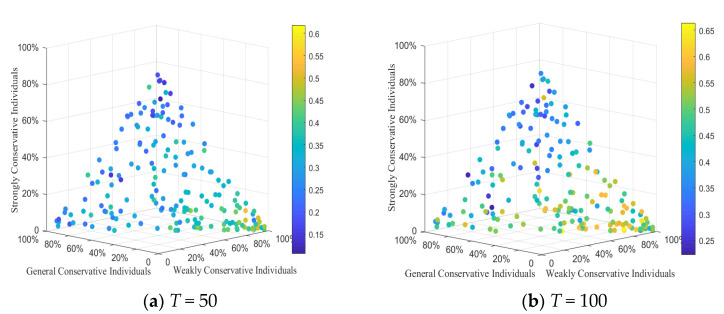
Attitude polarization of three conservative individuals at random proportion.

In order to better analyze the influence of the distribution of three types of individuals in the network on the public opinion reversal, on the basis of the above simulation, 200 experiments were conducted, respectively. Each experiment randomly generated different quantities of the three types of individuals and formed different social networks (i.e., weakly conservative individuals account for 42%, generally conservative individuals account for 28%, strongly conservative individuals account for 30%). The negative polarizability and positive polarizability generated under each combination were recorded, and the four-dimensional scatter diagram of polarization (color represented one-dimensional) was drawn, as shown in Figure 18. Each scatter represents one simulation result, and the *x* axis, *y* axis and *z* axis represent the proportion of weakly conservative individuals, generally conservative individuals and strongly conservative individuals, respectively. Scatter color indicates polarization rate: shallow color means higher polarization rate, and vice versa, which is shown in the color scale on the right. The concrete results are shown in Figure 18.

Figure 18 shows the attitude polarization of three conservative individuals at random proportion. As can be seen from Figure 18, at *T* = 0~50, under the influence of negative external information, the scatter color becomes lighter and lighter as the proportion of weakly conservative individuals increases, indicating that the negative polarization at this time increases with the number of weakly conservative individuals. At *T* = 50~100, under the influence of positive information, with the increase of weakly conservative individuals, the larger number of light scatters in the figure illustrates the higher positive polarization rate. It demonstrates that, due to the increase of weakly conservative individuals, public opinion reverses greatly, and individual conservation obviously impacts on public opinion reversal.

**Figure 19:**
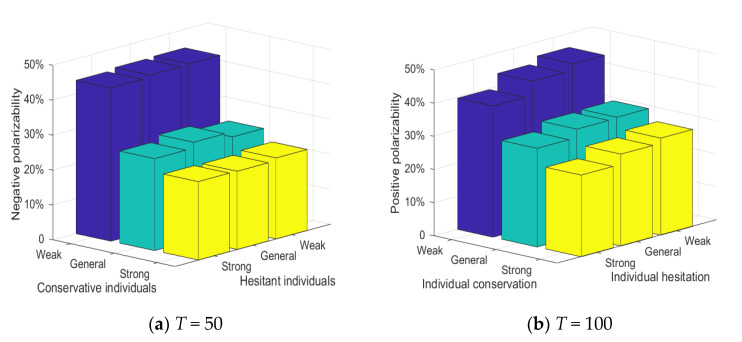
Relationship between three conservative individuals, hesitant individuals and attitude polarization.

Figure 19 shows the relationship between three conservative individuals, hesitant individuals and attitude polarization. Each cube in the figure represents the attitude polarization generated by a combination of i.e., (weak conservative, strong hesitant), (strong conservative, weak hesitant). Meanwhile, in order to ensure the consistency of simulation conditions, the proportion of each conservative individual and hesitant individual in the network is set to be 100% in the experiment. It can be seen from Figure 19 that at *T* = 0~50, when all the individuals in the group are weakly conservative, the negative polarizability is generally higher and less affected by the change of individual hesitation. At *T* = 50~100, when all the individuals in the group are weakly conservative, the positive polarizability is generally higher. With the decrease of individual hesitation, the positive polarizability presented a small increase. This shows that individual conservatism is an important factor affecting the polarization and public opinion reversal, and individual hesitation will only play a small role in the process of public opinion reversal.

#### 4.3.4. Analysis of the Combination of Factors Influencing the Public Opinion Reversal

Through the above simulation analysis, it can be seen that there are many factors affecting public opinion reversal. However, in the real world, due to the urgency of the development of online public opinion, it is usually necessary to focus on the key links, so as to respond to the further outbreak of public opinion in a timely manner. Therefore, it is more in line with the practical requirements to find out the most critical among the many factors affecting public opinion reversal. This section will find out the key factors affecting the polarization and reversal of public opinion by analyzing the combination of factors.

**Figure 20:**
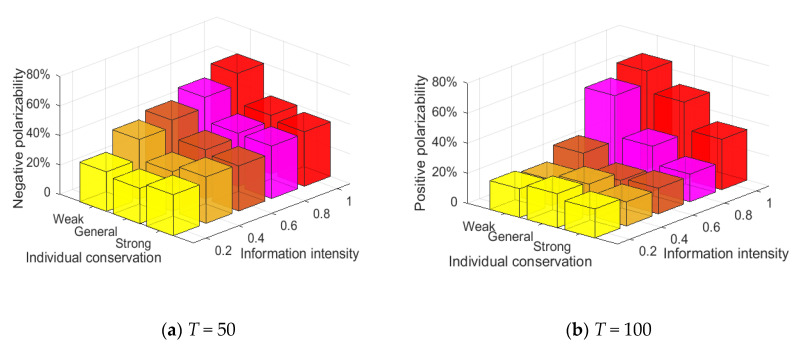
Relationship between information intensity, individual conservation and attitude polarization.

Figure 20 shows the relationship between information intensity, individual conservation and attitude polarization. As can be seen from Figure 20, negative polarizability increases to a certain extent with the increase of external information intensity at *T* = 0~50. When the network is all weakly conservative individuals, the negative polarizability is generally higher than the other two cases, indicating that individual conservation has an important impact on the polarization of viewpoints. At *T* = 50~100, when the external information intensity is small, the positive polarizability is generally low, indicating that there is no obvious public opinion reversal at this time. With the further enhancement of the external information intensity, the positive polarizability increases significantly, indicating that there is a relatively significant positive polarization and an obvious public opinion reversal at this time. Thus, individual conservation has a greater impact on the initial polarization state of public opinions, while the intensity of subsequent external intervention information has a greater impact on further public opinion reversal.

**Figure 21:**
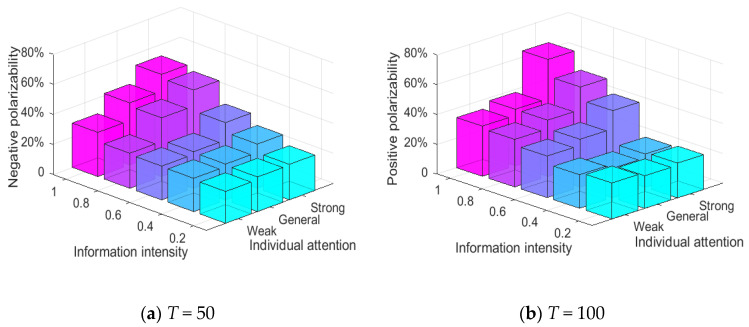
Relationship between information intensity, individual attention and attitude polarization.

Figure 21 shows the relationship between information intensity, individual attention and attitude polarization. As can be seen from Figure 21, at *T* = 0~50, the negative polarizability increases significantly, with the gradual enhancement of external information intensity and individual attention, indicating that under the influence of external information intensity and individual attention, the individuals in the group form an obvious consensus of views. At *T* = 50~100, the influences of external information intensity and individual attention are more obvious, that is, when both external information intensity and individual attention are relatively strong, the positive polarization rate changes significantly, forming a new round of positive polarization, which leads to a relatively obvious public opinion reversal. In addition, it can be seen from Figure 21 that, when the individual attention is weak, even a strong external information intervention will not produce a strong public opinion reversal. Therefore, it can be concluded that the intensity of external information and the strength of individual attention are the key factors affecting the polarization and reversal of public opinion, and the degree of individual attention has a greater impact on public opinion reversal.

**Figure 22:**
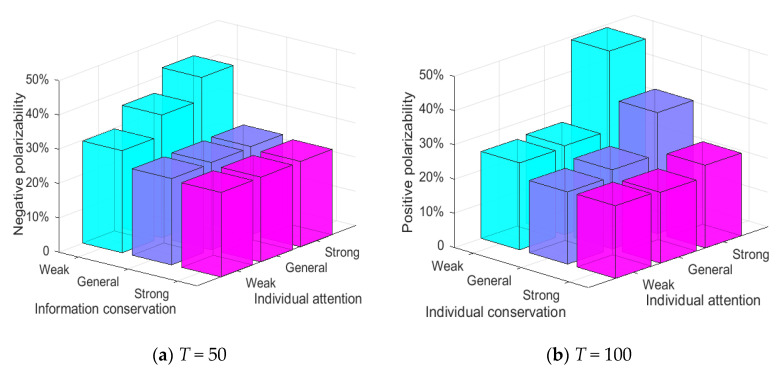
Relationship between conservation, individual attention and attitude polarization.

Figure 22 shows the relationship between conservation, individual attention and attitude polarization. As can be shown in this figure, at *T* = 0~50, when individuals are generally conservative individuals or strongly conservative individuals, the change of individual attention does not change negative polarizability, but when individuals are weakly conservative individuals, the negative polarizability grow significantly, suggesting that the degree of individual conservation has a key influence on public opinion polarization. At *T* = 50~100, when individual attention is weak, the fluctuation of individual conservation degree does not change the positive polarization rate, but when individual attention is generally strong, positive polarizability obviously enhances, suggesting that individual attention has a key influence in a new round of public opinion polarization. Thus, individual conservation plays a key role in the generation of initial public opinion polarization, while individual attention plays an important role in the subsequent public opinion reversal.

To sum up, individual conservation is the key factor affecting the initial public opinion polarization, while external intervention information intensity and individual attention are the key factors causing the public opinion reversal.

### 4.4. The Influence of Individual Interaction Mode on the Public Opinion Reversal

Netizens’ attitudes towards public events are not only influenced by external information and individual internal characteristics, but also by the views of their neighbors. Therefore, the analysis of the influence of the attitude of the surrounding individuals in the process of interaction is critical to the study of the polarization and reversal of public opinion. Based on this, the following section will analyze the process of public opinion reversal from the perspective of individual interaction.

#### 4.4.1. The Influence of Individual Interaction Times on the Public Opinion Reversal

In the process of individual interaction, the number of interactions between individuals and the surrounding nodes can usually be regarded as the depth of the communication of views between individuals. The deeper the communication of views between individuals is, the easier it is to reach a consensus, thus promoting the formation of stronger polarization and reversal of public opinions.

**Figure 23:**
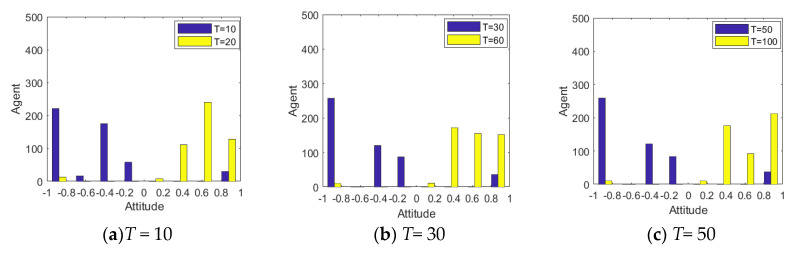

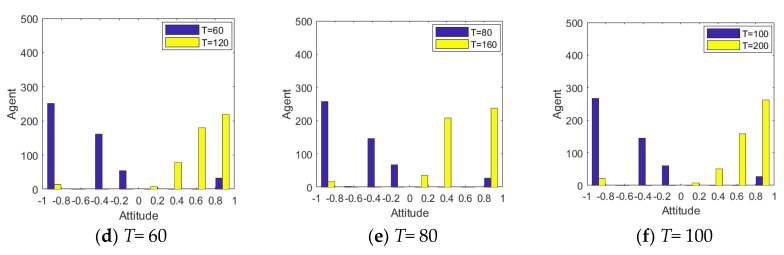
Distribution histogram of group attitude under different interaction times.

**Figure 24:**
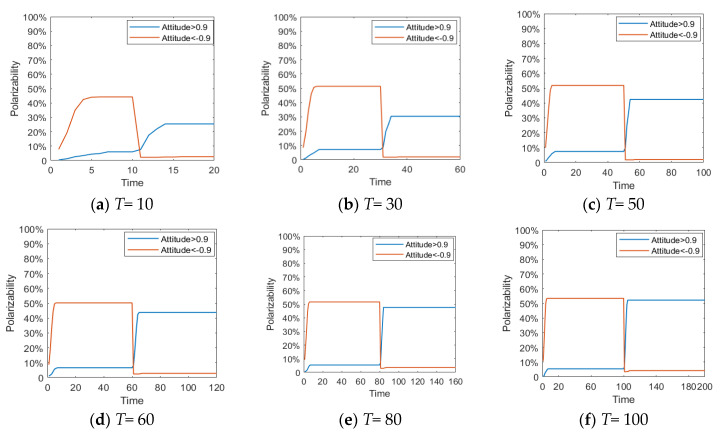
Curve of group attitude polarization under different interaction times.

Figure 23 and Figure 24 represent the distribution histogram of group attitude and curve of group attitude polarization under different interaction times, respectively. As can be seen from these figures, with the increasing interactions between individuals, the negative polarizability gradually tends to be stable after increasing to a certain extent, which indicates that under the influence of initial information, individuals can reach a consensus after a short period of interaction. Under the influence of the secondary information, the positive polarizability increases with the increase of interactions, and does not become stable until the number of interactions reaches about 100. This indicates that, under the influence of secondary information, it takes a longer time for individuals to interact with each other to form a new consensus.

#### 4.4.2. The Influence of the Number of Interaction Nodes on the Public Opinion Reversal

According to the interaction rules proposed in the paper, the node with an intimacy = 1 with agent *i* is defined as its interaction node. In the interaction process, the individual will update their own attitude according to the average attitude of the interaction node. In general, when an individual attitude fluctuates, the individual is more willing to listen to the friends’ opinions. The study on the number of interaction nodes can further analyze the influence of neighboring nodes on individual attitudes, and then study how to influence the polarization and reversal of public opinion through the interaction process between individuals. Here, the ratio of interaction nodes is changed (for example, agent *I* finds 30 interaction nodes in the network and randomly selects 10%, 20%, 30% to describe changes in the number of interacting nodes.)

**Figure 25:**
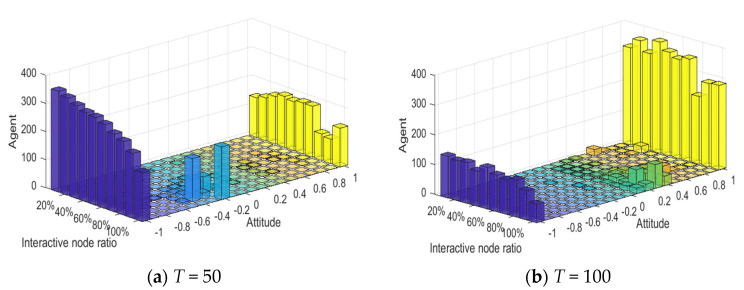
Evolution diagram of group attitude under different interaction node proportions.

Figure 25 shows the evolution diagram of group attitude under different interaction node proportions. As can be seen in Figure 25, at *T* = 0~50, with the increase of the proportion of interaction nodes under the influence of negative information, the number of agents with an individual attitude value around −1 gradually decreases. At *T* = 50~100, the number of agents with an individual attitude value around 1 also decreases, under the influence of the secondary information. This shows that, after combining the opinions of the interaction nodes, the individual attitude tends to be more rational, and the number of extreme individuals in the group gradually decreases, which is consistent with the reality. This phenomenon indirectly proves why the enhancement of individual conservation leads to the reduction of the number of extreme individuals. The reason for this lies in the fact that the enhancement of individual conservation leads individuals to listen to the opinions of friends, thus making their attitude more rational.

### 4.5. The Impact of Network Topology Structure on Public Opinion Reversal

The spread of external information and the interaction of individual opinions are carried out through social networks, which are the basis for generating online public opinions. However, real social networks are extremely complex, and it is difficult to use a certain network to truly reproduce them. Based on this, in order to more reasonably analyze the impact of network topology on the process of public opinion reversal, a comparative analysis of the process of public opinion reversal is made here, based on the network structure with different parameters. The parameters are set as follows: *I*^1^ = −0.6, *I*^2^ = 0.8, *α* belongs to *N*~(0.9,0.4), 80%weakly conservative individuals, 10% generally conservative individuals, 10% strongly conservative individuals and proportions of the three individuals are uniformly distributed.

Different network structures have a different node connection mechanism and growth mechanism, and, to some extent, they also represent different information exchange and transmission modes among groups, which have a great impact on the spread of information, the interaction of views, the polarization and reversal of public opinion. Based on this, this section studies the impact of network structure on the public opinion reversal through the comparative analysis of simulation between the BA scale-free network and the fully connected network [29], the Watts–Strogatz (WS) small world network [30] and the Erdos–Renyi (ER) (proposed by Erdos & Renyi) random network [31]. Different network topology parameters are shown in Table 3.

**Figure 26:**
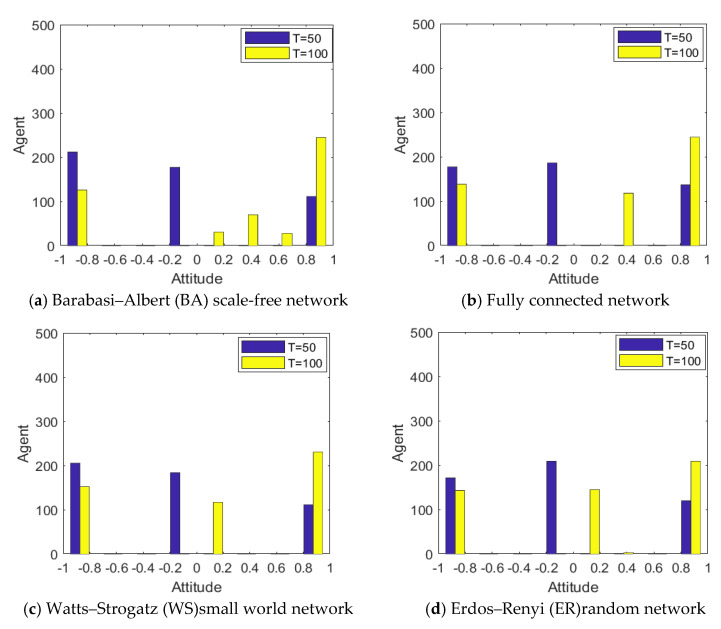
Distribution histogram of group attitude under different networks.

**Figure 27:**
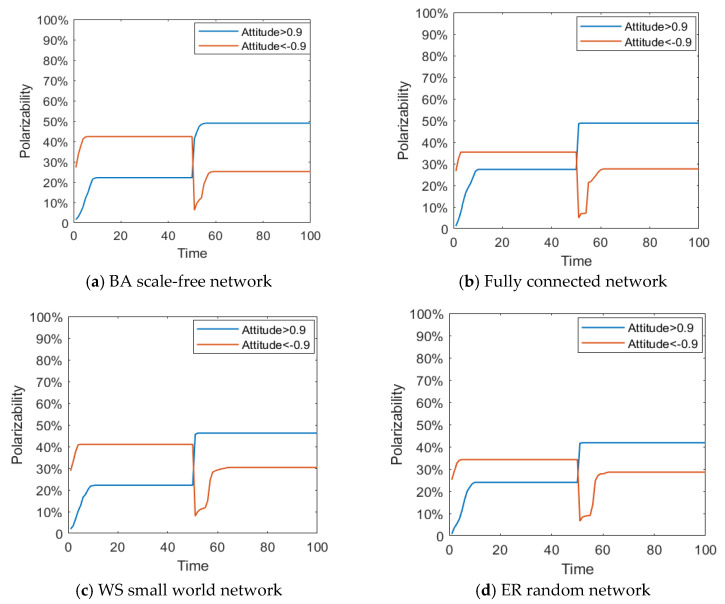
Curve of group attitude polarization under different networks.

Figure 26 and Figure 27 represent the distribution histogram of group attitude and curve of group attitude polarization in the BA network, fully connected network, WS small world network and ER random network, respectively. From Figure 26 and Figure 27, compared with the group attitude distribution and polarizability curve under the BA network, the group attitude distribution and polarizability slightly change in different network structures. Firstly, in the fully connected network, the negative polarizability is relatively low under the influence of the initial information, indicating that the network structure at this time inhibits the initial polarization of attitude to some extent. Secondly, in the WS small-world network, the attitude distribution and polarizability in the group do not change significantly, which is consistent with the evolution results under the BA network. Finally, in the ER random network, the negative polarizability is relatively low under the influence of the initial information, and the positive polarizability is also low under the influence of the secondary information, indicating that the network structure at this time inhibits the initial polarization of attitudes and the later public opinion reversal to some extent. However, on the whole, under the change of external information and individual internal characteristics, public opinion reverses, to different degrees, in the four network structures, which indicates that the network structure has a certain influence on the process of public opinion reversal, but that this influence is relatively limited.

## 5. The Real Case Study

This section selects a typical case “Liu Qiangdong (Richard Liu) sexual assault” (https://s.weibo.com/weibo/%25E5%2588%2598%25E5%25BC%25BA%25E4%25B8%259C%25E6%2580%25A7%25E4%25BE%25B5?topnav = 1&wvr = 6&b = 1) to verify the network public opinion reversal model established in this paper.

On 2 September 2018, Liu Qiangdong, the CEO of JD.com was arrested on suspicion of sexually assaulting a female college student in Minnesota, the United States. A photo of Liu’s arrest went viral online. On 3 September, the number of relevant media reports on the incident exceeded 6000, and netizens condemned with one accord. On 21 December 2018, Minneapolis prosecutors decided not to charge Mr. Liu with sexual assault. The news of Liu Qiangdong’s acquittal spread on the Internet again. At this time, the focus of netizens’ comments turned to the assaulted woman, trade war, etc., and the overall public opinion was greatly reversed from the first stage. Since the event lasted for more than three months, and the man involved was a public figure with strong influence, the release of information relevant to the event could always cause a strong response from netizens, and with the continuous change of external information, a series of public opinion reversal was formed.

**Figure 28:**
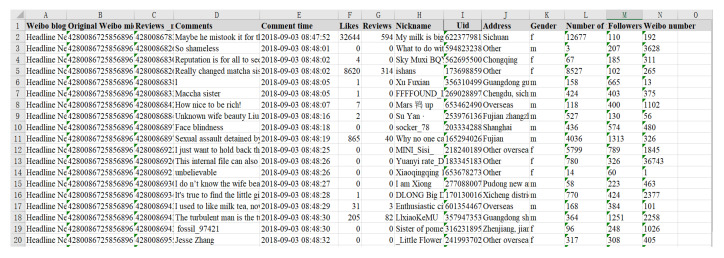
Relevant comments.

To analyze this public opinion reversal process, this paper selects relevant news from Toutiao, Btime, the Beijing News, the Paper released on 3 September 2018 and on 22 December chooses 6000 comments from 30,000 Weibo comments and likes. JIEBA (“Jieba” (Chinese for “to stutter”) Chinese text segmentation: built to be the best Python Chinese word segmentation module.) and the emotion dictionary were used to score their emotions, and the emotion value of each Weibo comment was quantified. In spite of limited amount of data obtained, according to the six degrees of separation theory [32], the statistical results of these user data can largely reflect some universality of Weibo user behavior. The dates 3 September and 21 December were defined as the first and second stages of the event, respectively. The form of results is shown in Figure 28.

**Figure 29:**
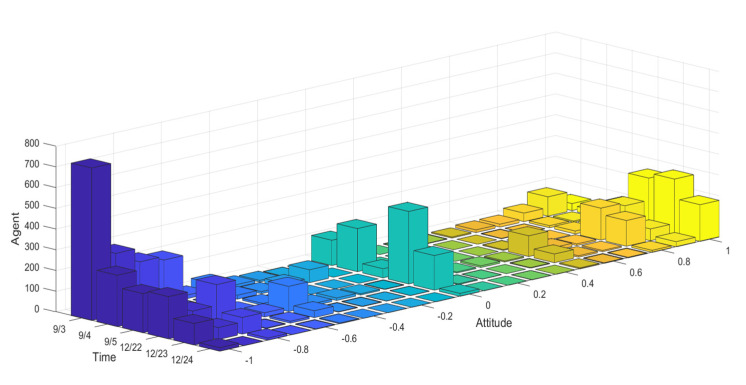
Diagram of public opinion evolution.

**Figure 30:**
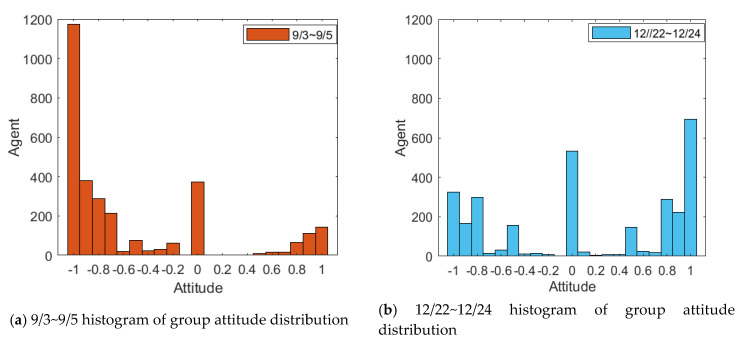
Histogram of group attitude distribution.

Due to the overall negative trend of the Weibo comments on the event, during the emotional rating process, some negative comments from subjects in other dimensions were handled. For example, trade war, being framed, being trapped and other comments with positive attitudes were classified into positive words. The scoring results of Weibo comments on 3–5 September, and 22–24 December were arranged in the order of the release time of comments, and a histogram of the evolution of public opinion and the distribution of group attitudes was generated. The results are shown in Figure 29 and Figure 30.

Figure 29 and Figure 30 represent the diagram of public opinion evolution and histogram of group attitude distribution. Comments on 3 September, the initial time of the outbreak of public opinion, were numerous, and most netizens held extremely negative views and condemnation, forming the “one-sided” criticism, and only a minority of netizens expressed support or sympathy. In the following two days, due to the decline of the event, the number of comments decreased, but the overall public opinion was still relatively negative. However, after 22 December, due to the release of “acquittal” and other related news, netizens’ comments changed to a certain extent. Most netizens’ attitude turned positive and neutral. Compared with the previous “one-sided” negative comments, the public opinion of the event reversed. However, as the whole event is relatively negative, it can be seen from the figures that the degree of public opinion reversal is not large.

**Figure 31:**
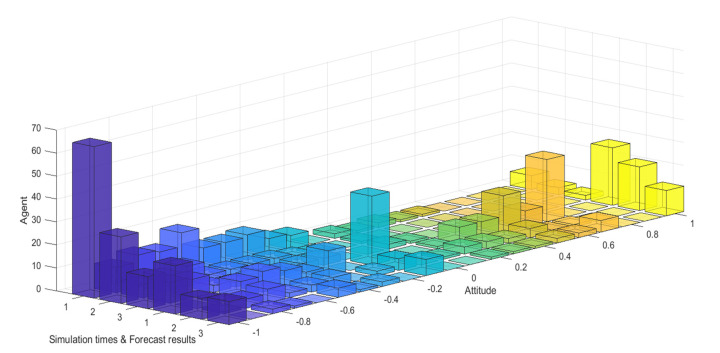
Simulated public opinion evolution diagram.

**Figure 32:**
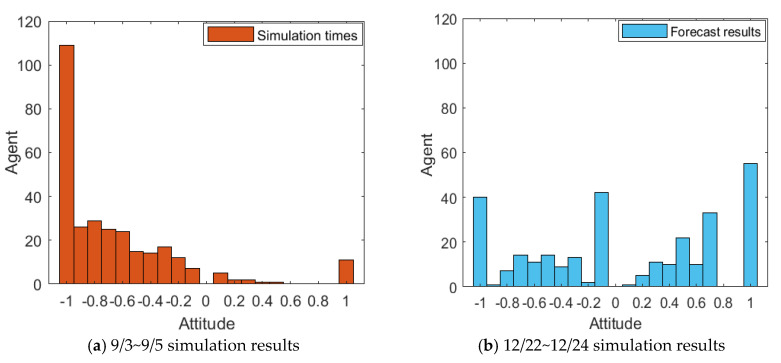
Simulated group attitude distribution histogram.

The event is simulated according to the public opinion reversal model proposed in this paper. Due to the large scale of case data and the comprehensive consideration of visualization, the number of simulation network nodes is set to 300. As a well-known entrepreneur with certain influence, Liu Qiangdong is generally well recognized by the public. Therefore, *α* is subject to *N*~(0.9,0.4) and mapped to [0,1]. The proportion of weakly conservative, generally conservative and strongly conservative individuals in the group was 50%, 40% and 10%, respectively. The proportion of individuals with weak hesitation, general hesitation and strong hesitation weakly in the group obeys uniform distribution, and other parameters are set as follows: *d*_1_ = 0.18, *d*_2_ = 0.68, *µ* = 0.48, *β* = 0.2, *I*^1^ = −0.8, *I*^2^ = 0.8. The simulation results are shown in Figure 31 and Figure 32.

Figure 31 and Figure 32 represent the simulated public opinion evolution diagram and group attitude distribution histogram of “Liu Qiangdong sexual assault” by the model proposed in this paper. In order to better simulate the process of public opinion reversal, three simulations were carried out on public opinion evolution during 9/3~9/5, whose results were corresponding to the public opinion evolution during 12/22~12/24 (such as: simulation times 1 corresponds to case 9/3, forecast results 1 corresponds to case 12/22). From Figure 31 and Figure 32, three model simulations presented good state of public opinion evolution in the first stage (9/3~9/5), and the evolution trend of public opinion is consistent with the evolution of the case data on the whole, simulated group distribution state is consistent with case data distribution, the simulated prediction results are basically consistent with the actual evolution results in the second stage (12/22~12/24). This shows that the model proposed in this paper can well simulate the evolution of real public opinions and predict the evolution results of further public opinion reversal. Therefore, the model proposed in this paper is reasonable and effective and of practical significance.

**Figure 33:**
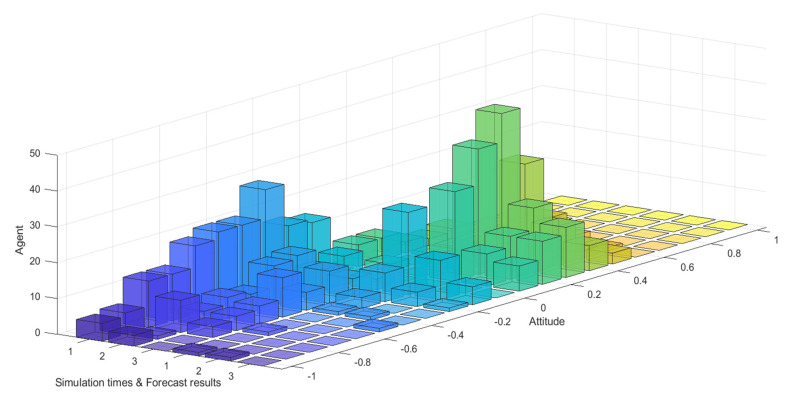
Simulated public opinion evolution diagram based on the literature [14].

**Figure 34:**
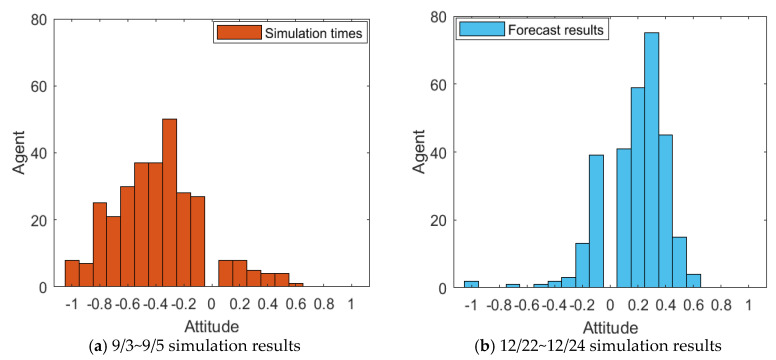
Simulated group attitude distribution histogram based on the literature [14].

In order to further compare the differences with other models, the public opinion reversal model proposed in the literature [14] was used to simulate the “Liu Qiangdong sexual assault”, and the advantages of the model were further illustrated by comparing the simulation effects of the two models on cases. The simulation results of the model in the literature [14] are shown in Figure 33 and Figure 34.

Figure 33 and Figure 34 represent the simulated public opinion evolution diagram and group attitude distribution histogram of “Liu Qiangdong sexual assault” by the model proposed in the literature [14], respectively. As can be seen in Figure 33 and Figure 34, the public opinion reversal model does not simulate the extreme individuals in the evolution of public opinion. In the process of overall public opinion evolution, the number of extreme individuals is very small, which is inconsistent with the reality. In addition, although the model reflects the state of public opinion reversal on the whole, it differs greatly from the actual result of the reversal and the state of views distribution. Therefore, the public opinion reversal model proposed in this paper is more reasonable.

## 6. Conclusions

In order to reveal the mechanism of network public opinion reversal, this paper constructs a public opinion reversal model by combining external intervention information and individual internal characteristics, and then it simulates and analyzes the influence of information intensity, individual attention, individual conservation, individual interaction pattern and network topology on the process of public opinion reversal.

The following conclusions are obtained through the simulation experiment:

(1) The intensity of external information, especially the intensity of secondary information, is an important factor leading to the public opinion reversal [14], and information intensity will affect the direction and degree of the reversal. Therefore, relevant institutions should strengthen the monitoring of online public opinion, and promptly publish authoritative information to prevent multiple fluctuations and the public opinion reversal.

(2) In terms of individual internal characteristics, the change of individual attention is the key factor that leads to further reversal. Individual conservation has a stronger influence on the initial polarization state of public opinion. Correspondingly, individual hesitation has a minor influence on the polarization and reversal of public opinion. Therefore, relevant institutions should strengthen the publicity and guidance of public opinion, and improve the public’s ability to recognize external information.

(3) When the external information is strong, the group opinion tends to produce strong polarization and reversal of public opinion. However, through simulation, it is found that, when individual conservation is strong or individual attention is weak, even if external information is strong, there is still no obvious public opinion reversal phenomenon. Therefore, in the process of public opinion monitoring, relevant organizations should strengthen the recognition of the nature of public opinion.

However, this paper still has the following limitations and it opens the possibility of further studies:

(1) According to the actual case, the derivative nature of the topic is a common phenomenon in public opinion propagation, which means there exist multi-dimensional attitude interactions. However, the model proposed in this paper cannot reasonably explain this situation. Therefore, the model needs to be extended to cover multi-dimensional attitude reversals in subsequent studies.

(2) As the spread of hot social events is usually dynamic, with the release of external information, the network nodes participating in the event discussion will increase or decrease, so it is necessary to consider the increase and decrease mechanism of nodes in the network [33,34] and study the phenomenon of public opinion reversal in the dynamic network.

## Figures and Tables

**Table 1:** Parameters involved in the model.

*α_i_*	Attention degree of *i*
*k_i_*	Conservation degree of *i*, the larger the *k* is, the more conservative the individual is
*h_i_*	Hesitation degree of *i*, the small *h* is, the more willing individual is to adopt new information
*d*_1_	Assimilation effect distance
*d*_2_	Repulsion effect distance
*µ*	Assimilation parameter
β	Repulsion parameter
*j^′^*	The node whose intimacy is 1 with *i*, that is interaction node of agent *i*

**Table 2:** Variables involved in the model.

$p_{i}$	The probability that node *i* receives the information
*I^n^*	Information intensity of the *n*th information
$O_{i}^{n}$	The amount of information perceived by node *i* in the presence of the *n*th information, that is the amount of information the individual perceives
$d_{ij}$	The distance between node *i* and node *j*
$q_{ij}$	The intimacy between node *i* and node *j*
$x_{i}^{n}$	The attitude value of node *i* in the presence of the *n*th information
${\dot{x}}_{i}^{n}$	The attitude value of node *i* after interacting with neighboring nodes
$X_{j^{\prime }}^{n}$	The average attitude value of node *j**^′^***

**Table 3:** Different network topology parameters.

Name	Average Path Length	Clustering Coefficient	Average Degree
BA scale free network	2.3582	0.09122	18.948
Fully connected network	2.3808	1	499
WS small world network	2.3808	0.081902	8
ER random network	2.3808	0.099496	49.52
